# The psychosocial burden of visible disfigurement following traumatic injury

**DOI:** 10.3389/fpsyg.2022.979574

**Published:** 2022-08-30

**Authors:** David B. Sarwer, Laura A. Siminoff, Heather M. Gardiner, Jacqueline C. Spitzer

**Affiliations:** ^1^Department of Social and Behavioral Sciences, College of Public Health, Temple University, Philadelphia, PA, United States; ^2^College of Public Health, Temple University, Philadelphia, PA, United States

**Keywords:** vascularized composite allotransplantation, psychosocial issues, visible disfigurement, stigma, traumatic injuries

## Abstract

Hundreds of thousands of individuals experience traumatic injuries each year. Some are mild to moderate in nature and patients experience full functional recovery and little change to their physical appearance. Others result in enduring, if not permanent, changes in physical functioning and appearance. Reconstructive plastic surgical procedures are viable treatments options for many patients who have experienced the spectrum of traumatic injuries. The goal of these procedures is to restore physical functioning and reduce the psychosocial burden of living with an appearance that may be viewed negatively by the patient or by others. Even after receipt of reconstructive procedures, many patients are left with residual disfigurement. In some, disability and disfigurement may be so profound that individuals are candidates for vascularized composite allotransplantation (VCA) procedures, i.e., the transplantation of a vascularized human body part containing multiple tissue types (skin, muscle, bone, nerves, and blood vessels) as an anatomical and/or structural unit. This narrative review paper summarizes the literature on the psychosocial burden experienced by those who have visible disfigurement. While many of these individuals experience stigma and discrimination, relatively few studies have employed a stigma framework to understand the psychosocial sequelea. This paper briefly addresses this framework. Last, particular focus is given to the psychosocial issues of individuals with particularly severe injuries who are potential candidates for VCA procedures.

## Traumatic injury

Traumatic injuries, both those experienced unintentionally through accidents and those that are violence-related, are estimated to constitute approximately 8% of deaths around the world each year (World Health Organization [WHO], 2021). In 2020 in the United States, traumatic injuries accounted for over 270,000 deaths (Centers for Disease Control and Prevention [CDC], 2022). Over 3 million Americans are estimated to experience non-fatal injuries per year (American Association for the Surgery of Trauma, n.d.). These traumatic injuries are believed to account for an estimated 10% of the life years that an individual lives with a disability (World Health Organization [WHO], 2021). The health care costs associated with acute and chronic treatment of persons who have suffered traumatic injuries is staggering. In 2019, the cost of injuries from fatal and non-fatal injuries was $4.2 trillion. This cost includes medical care, work loss, statistical, and quality of life losses (Peterson et al., 2021). The experience of a traumatic injury increases the risk of mental health issues, substance misuse, chronic disease such as cardiovascular disease and type 2 diabetes, as well as poverty, crime, and violence (Pacella et al., 2013; Hughes et al., 2017). There is a dose response between the lifetime experience of traumatic events and the increased odds of developing significant health issues over time (Scott et al., 2013).

The experience of traumatic injuries is particularly high among specific groups of individuals. Severe automobile accidents, injuries from firearms, workplace injuries, fires, and unsuccessful suicide attempts can leave individuals with traumatic injuries. The number of individuals who suffer these injuries around the world each year is difficult to calculate; more reliable numbers are available from selective groups. For example, in 2006, over 8,000 active duty American military personnel suffered bodily injuries resulting in hospitalization (Jones et al., 2010). Between 2003 and 2011, approximately 7,200 American soldiers stationed in Iraq and Afghanistan sustained significant head and neck injuries (Brennan, 2015). During Operation Iraqi Freedom, wrist, hand, and finger injuries accounted for 28.7% of all extremity injuries (Dougherty et al., 2009). Approximately 30% of military personnel who sustained an extremity injury (either upper or lower) also experienced an injury to the face. While the nature of these injuries is often severe, the survival rate from them is high (Dougherty et al., 2009).

The experience of trauma, and the risk of the most profoundly deleterious effects, is highest among those individuals from underserved groups, making traumatic injuries a significant public health issue (Merritt and Benningfield, 2019). As with other major public health issues, social determinants of health increase the risk of both experiencing a traumatic injury and potentially threaten a successful course of physical and/or mental health treatment following the experience.

## Medical care of traumatic injuries

The course of recovery from a traumatic injury is influenced by the delivery of high quality health care at all points in the treatment process. Quality emergency care can reduce the risk of fatality, disability, and psychosocial adjustment. High quality rehabilitation services and the promotion of community inclusion by removing barriers to social and economic participation can ensure that people who experience disability following a traumatic injury enjoy the fullest life possible.

Plastic surgeons are often centrally involved in the care of traumatic injuries. The American Society of Plastic Surgeons (2021), for example, reported that approximately 6.9 million reconstructive surgical procedures were performed in 2020. The most common were tumor removal, laceration repair, scar revision, and maxillofacial and hand procedures. In addition to treating functional issues, these procedures have a major goal of restoring physical appearance to an approximation of normal.

Advances in medical and surgical care have improved the survivability and rehabilitation of individuals who have suffered severe, traumatic injuries (Holcomb et al., 2006; Eastridge et al., 2012). However, even the most successful reconstructive surgical procedures leave patients with some degree of residual deformity. For example, an individual who falls off of a bicycle and suffers facial fractures and lacerations may have visible scarring for the rest of her life. In the case of limb loss, some individuals have the option of prosthetics. Advances in prosthetic technology has improved the physical functioning and quality of life of many individuals with disabilities (Magee et al., 2011). Some individuals show tremendous resilience in their ability to adapt to living with profound disability and disfigurement. Others, unfortunately, are unable to benefit from prosthetic arms and hands (Grunert, 2006; DeBerard and Goodson, 2013). The resulting functional limitations often reduce their ability to perform work-related tasks and negatively impact self-esteem, body image, and quality of life. Many of these individuals suffer with concomitant and significant psychosocial burden in conjunction with the residual disability and disfigurement (Grunert, 2006; Magee et al., 2011; DeBerard and Goodson, 2013).

## Psychological response to traumatic injury

Individuals can experience a wide range of psychological reactions to a traumatic injury, regardless of whether or not the injury results in permanent changes to one’s body and physical appearance. From a psychiatric perspective, a traumatic event, whether it involves a traumatic injury or not, must involve actual or threatened death or serious injury or sexual violence. The individual experiencing the event also must experience intense fear, helplessness, or horror (American Psychiatric Association [APA], 2013).

Two psychiatric diagnoses of relevance to traumatic events are acute stress disorder (ASD) and posttraumatic stress disorder (PTSD). Both ASD and PTSD are diagnosed in persons who experienced (or witnessed) a traumatic event and experience symptoms including re-experiencing the traumatic event in memories, intrusive thoughts, dreams, or flashbacks, avoiding reminders of the event (including medical or mental health treatment visits), and increased feelings of arousal, anxiety, and depression. In ASD, these symptoms begin within 4 weeks of event and last for less than 1 month. PTSD may develop from ASD, but symptoms last for longer than 1 month. In both disorders the symptoms must cause significant distress or impairment.

Not everyone who experiences a traumatic injury experiences ASD and/or PTSD (Resick et al., 2008). While men are exposed to more trauma, women have higher rates of ASD and PTSD (Resick et al., 2008). While it can be difficult to predict why some individuals develop these traumatic stress reactions and others do not, some variables have been identified (Magee et al., 2011). Several social determinants of health (poverty, neighborhood crime), mental health problems in others in the home, and a lack of social support are associated with less positive reactions to trauma (Schroeder et al., 2021). The experience of adverse childhood experiences includes situations of physical, emotional, or sexual abuse, physical and emotional neglect, as well as exposure to household stressors including family member’s substance use, mental illness, incarceration, intimate partner violence, divorce/separation, or death also is associated with greater psychological difficulties with later life trauma (Felitti et al., 1998).

Depression is common among survivors of traumatic injuries. For those who have a visible disfigurement from their injuries, social anxiety disorder may also be observed. It is defined as a marked and persistent fear of one or more social or performance situations in which the person is exposed to unfamiliar people or to possible scrutiny by others (American Psychiatric Association [APA], 2013). The individual fears that he or she will act in a way (or show anxiety symptoms) that will be humiliating or embarrassing. Exposure to the feared social situation almost invariably provokes anxiety, which may escalate to a panic attack. The avoidance, anticipation, or distress in the feared situation interferes significantly with the person’s typical, daily functioning.

## Psychosocial issues in military veterans

There is a sizable literature on the mental health issues of military personnel who have suffered traumatic injuries or witnessed traumatic events (Hoge et al., 2006; Seal et al., 2007; Kessler et al., 2014; Hom et al., 2017; Kraus, 2017). Individuals who serve in the military are found to have high rates of depression, anxiety disorders, substance abuse, and suicidality following discharge (Hoge et al., 2004; Eisen et al., 2012; Gadermann et al., 2012; Sirratt et al., 2012; Fisher, 2015). Approximately 20% of American military personnel who returned from the Iraq war screened positive for mental health disorders, including PTSD, anxiety, or depression (Hoge et al., 2004; Eisen et al., 2012). The rates of these conditions are even higher among those who were engaged in combat as well as those who experienced and/or witnessed a traumatic event that resulted in injury to themselves or fellow soldiers (Clemency Cordes et al., 2016).

Mental health issues may be of particular concern to veterans who have suffered disfiguring injuries. Approximately 40% of individuals exposed to trauma receive a diagnosis of PTSD (O’Donnell et al., 2003). The rate of PTSD among those who suffered debilitating injuries is believed to be even higher (Fisher, 2015). The permanent and visible nature of these disfiguring injuries serves as a daily reminder of their trauma and is believed to be a stimulus to the development and/or maintenance of PTSD (Van Loey et al., 2003).

Depression also is common. For example, nearly 25% of veterans who suffered traumatic limb loss have been diagnosed with a mood disorder (Reiber et al., 2010). Either as a consequence of or concomitant to depression, substance abuse rates among military veterans are high (Seal et al., 2011). Suicidality is a particularly troubling concern; estimates suggest that approximately 20 American military veterans attempt to end their own lives daily (Department of Veterans Affairs Office of Suicide Prevention, 2016). The rates of these issues among those appearance altering disfigurement is likely even greater.

Combat related injuries that profoundly disfigure military personnel likely have the potential to produce a traumatic brain injury (TBI). In the last several years, the occurrence of TBI among military personnel, as well as the general public, has received increased research and clinical attention. Approximately 440,000 military personnel have experienced a TBI since 2000 (Traumatic Brain Injury Center of Excellence [TBICoE], 2021). In contrast, approximately 1 million civilian Americans are believed to experience a TBI annually; 50,000 die from these injuries and approximately 8–10% have chronic symptoms (Centers for Disease Control and Prevention [CDC], 1999).

While some of these injuries are mild and resolve over time, others have long-term effects. Individuals with TBIs may experience neurocognitive deficits, PTSD, depression, anxiety, substance abuse, and suicidality (Hoge et al., 2008; Litz and Schlenger, 2009; Bryant et al., 2010; Carlson et al., 2011; Mallya et al., 2015; Haabauer-Krupa et al., 2017; Swan et al., 2018). Persistent post-concussion symptoms have been reported in as many 85% of veterans who experienced a TBI during wartime service (Morissette et al., 2011). Individuals with long-lasting, unremitting impairments often report vestibular symptoms, such as dizziness, as well as postural and gait disturbance (Akin and Murnane, 2011; Baldassarre et al., 2015; Howell et al., 2015; Leddy et al., 2015). Other visual-vestibular symptoms that persist beyond the acute period include sensitivity to visual motion and deficits in oculomotor function (Ciuffreda et al., 1996; Kapoor and Ciuffreda, 2002; Heitger et al., 2009; Capo-Aponte et al., 2012; Mucha et al., 2014; Wright et al., 2015; McDevitt et al., 2016; Wright et al., 2017; Cheever et al., 2018).

These symptoms also are associated with impairments in psychosocial functioning (Bryant, 2008; Hoge et al., 2008; Wares et al., 2015). As noted above, PTSD is common in military veterans, although the TBI-PTSD relationship remains somewhat unclear (Carlson et al., 2011; Haabauer-Krupa et al., 2017). The prevalence of PTSD in military veterans is commensurate with the rate of TBI, with over 400,000 Veterans being seen for potential or provisional PTSD at Veteran Affairs facilities (Department of Veterans Affairs et al., 2017). TBI and PTSD share several symptoms, including concentration and/or memory loss, depression, irritability/anger, anxiety, as well as dizziness and loss of balance (Kennedy et al., 2007; Walker et al., 2014; Storzbach et al., 2015; Wares et al., 2015). Further complicating the relationship is the observation that TBI often occurs as a result of a traumatic event, a prerequisite for the diagnosis of PTSD (Servatius et al., 2017).

## Physical appearance and body image

The experience of a traumatic injury that results in visible disfigurement likely comes with additional psychosocial burden. Over the past 50 years, social psychologists have created a sizable body of research on the role of physical appearance in daily life. This research has repeatedly demonstrated that individuals who are less physically attractive are judged and treated less favorably than those who are more attractive (Eagly et al., 1991; Etcoff, 2000; Langlois et al., 2000; Sarwer and Spitzer, 2012a; Swan et al., 2018). For example, less attractive individuals are rated as being less intelligent, friendly, and kind than those who are more attractive.

Studies of individuals with facial and body disfigurement have replicated these findings (Tobiasen, 1987; Rankin and Borah, 2003; Mojon-Azzi et al., 2008; Masnari et al., 2013; Jamrozik et al., 2019). These results are consistent with the tenants of social-cognitive theory which postulates that portions of an individual’s knowledge acquisition, including understanding of the dynamics of social situations, results from observing others in the context of social interactions (Bandura, 1986). The theory states that when people observe an individual performing a behavior, and witness the results of that behavior, they remember the sequence of events and use this information to guide subsequent behaviors.

In the case of visible disfigurement, we learn from watching the behaviors of others that persons who look different should be treated different. In the case of those who are disfigured, that treatment is less favorable. Persons with facial disfigurement are rated as less attractive and assumed to have less positive personality traits as compared to those who are less disfigured or non-disfigured (Jamrozik et al., 2019). This has recently been described as “What is Anomalous is Bad,” where those with facial and body disfigurement are believed to have less positive personality traits and are more likely to engage in non-positive behavior (Workman et al., 2021). This is in contrast to the long standing “What is Beautiful is Good” bias (Dion et al., 1972). From a recent study using fMRI, it appears that these negative responses are “hardwired” into the occipito-temporal cortex as well as anterior cingulate cortex of the brain (Hartung et al., 2019), which may explain the particularly negative reactions that facial disfigurement illicits (Stone and Potton, 2019; Rasset et al., 2022). Encouragingly, plastic surgical procedures that minimize disfigurement result in more positive perceptions of individuals with disfigurement (Mazzaferro et al., 2017; Jamrozik et al., 2019).

At the same time, studies have suggested that perceptions of our own appearance, otherwise known as body image, play a significant role in quality of life and self-esteem (Sarwer and Spitzer, 2012a; Sarwer and Polonsky, 2016). In studies of individuals without visible disfigurement, there is either no relationship or a weak relationship between an individual’s objective appearance and their subjective body image (Sarwer et al., 2011). In studies of individuals with visible disfigurement, there is a more consistent relationship between the severity of disfigurement and degree of body image dissatisfaction (Rumsey and Harcourt, 2012; Crerand et al., 2017). Dissatisfaction with one’s appearance and body image is believed to be the motivational catalyst to pursue plastic surgical procedures to improve appearance (Sarwer and Crerand, 2008; Sarwer and Spitzer, 2012b).

Quality of life is a multifactorial construct that involves an individual’s degree of satisfaction and level of functioning in several core domains, including physical functioning, psychological well-being, as well as social and work role performance (Ware and Sherbourne, 1992). Body image is an important aspect of quality of life and is a highly relevant psychosocial construct for individuals who are disfigured. Along with pre-injury factors (such as premorbid psychopathology and social determinants of health), peri-traumatic factors (i.e., TBI, functional limitations, pain), and post-injury factors (i.e., social support), quality of life is believed to play a central role in adaptation to a disfiguring condition (Fauerbach et al., 2006; Block and Sarwer, 2013).

The psychosocial burden of living with a disfigured facial appearance cannot be underestimated. Much current understanding of this burden comes from studies of children born with cleft lip and/or palate or more profound craniofacial anomalies. In brief, children born with these conditions are at risk for significant psychosocial issues including depression, social anxiety, and reduced quality of life (Hunt et al., 2005; Demir et al., 2011; Broder et al., 2012; Tyler et al., 2013). Psychological functioning is related to individuals’ satisfaction with their facial appearance and body image (Moss, 2005; Magee et al., 2011; Crerand et al., 2015; Feragen et al., 2015). Adults with a visible disfigurement also report low self-esteem, body image dissatisfaction, depression, and anxiety (Pallua et al., 2003; Rumsey et al., 2004; Magee et al., 2011; Fingeret et al., 2012; Wisely and Gaskell, 2012; Akyol et al., 2013; Keeling et al., 2020). In a study of 458 adults with a range of visible disfigurements, 48% were judged to have symptoms of an anxiety disorder, and 28% had depression (Rumsey et al., 2004). In a separate study, 56% of patients with facial disfigurement were judged in need of mental health treatment (Strauss and Broder, 1991).

In summary, the current evidence base suggests that having a visible disfigurement may increase vulnerability to psychological distress – including depression, anxiety, and body image dissatisfaction. This distress may in part result from negative interactions with others, and/or the anticipation of unwanted attention due to their appearance. Anecdotal reports and a limited body of research suggest that stigma, if not outright discrimination, are common experiences among those with facial or body disfigurement. However, the resulting psychosocial and physical consequence of these experiences is less fully understood at present.

## Stigma and discrimination

Goffman (1963), arguably the world’s first scholar of stigma, began articulating categories and types of stigma over a half century ago. More contemporary scholars have defined stigma as the devaluation of social identities based on the recognition of difference based on some distinguishing characteristic (Dovidio et al., 2000). There are different levels of stigmatization. At the intrapersonal level, individuals may self-stigmatize their thoughts and feelings about a physical trait. The literature on the psychosocial burden of visible disfigurement detailed above falls at this level. At the interpersonal level, individuals may experience unwanted attention or treatment from others. At the structural level, individuals may encounter systematic, unfair treatment due to policies and practices that perpetuate stigma and discrimination.

A number of studies have documented the interpersonal stigma experienced by those with visible disfigurement (Rumsey and Harcourt, 2012). There are social consequences to having a disfigured appearance. As noted above, individuals with abnormal facial appearance are judged less positively as compared to those with normal facial characteristics (Tobiasen, 1987; Rankin and Borah, 2003; Mojon-Azzi et al., 2008; Masnari et al., 2013; Jamrozik et al., 2017). Individuals with facial disfigurement have been found to be the target of unwanted staring as well as negative appearance-related comments (i.e., teasing or bullying) from others (Sarwer et al., 1998, 1999; Turner et al., 1998; Strauss et al., 2007; Magin et al., 2008; Nishikura, 2009; Bonanno and Choi, 2010; Feragen and Borge, 2010; Lawrence et al., 2011; Bogart et al., 2012; Griffiths et al., 2012; Bogart, 2015; Sobanko et al., 2015; Halioua et al., 2017; Martin et al., 2017; Holland et al., 2019; Visram et al., 2019). The occurrence of this unwanted attention is associated with increased symptoms of depression and lower self-esteem (Rumsey et al., 2004; Crerand et al., 2017). Interestingly, some research has suggested that persons with facial disfigurement are seen less positively than those with other visible physical disabilities (Stevenage and McKay, 1999; Stone and Wright, 2012; Bogart et al., 2019).

Over the past two decades, research on stigma and discrimination of persons with obesity has blossomed (Puhl et al., 2020). This work can be used to further understand the interpersonal and structural levels of stigma in persons with visible disfigurement. Although obesity differs from visible disfigurement in several ways (e.g., obesity is generally viewed as more controllable than disfigurement and thus elicits more blame), it is similar in respect to being visible and impacting physical appearance. In a classic study, children ages 10–11 years were asked to rank order their liking of six children depicted with differences in appearance: obesity, facial disfigurement, wheelchair, crutches, missing hand, or no disability (Richardson et al., 1961). Across multiple groups, the child with obesity and the child with facial disfigurement were rated as the least preferred peer.

Weight-based stigma and discrimination is common. Data from the National Survey of Midlife Development in the United States indicated that rates of weight-based discrimination increased by 66% from 1995 to 2006 (Andreyeva et al., 2008; Puhl et al., 2008). This discrimination is not benign; it has been associated with mental health consequences. In a study of over 22,000 United States adults with overweight and obesity, over half who reported experiencing weight-based discrimination met criteria for at least one mood, anxiety, or substance use disorder; furthermore, the odds for meeting criteria for three or more comorbid disorders were 2.4 times higher than for individuals who had never experienced weight discrimination. Weight discrimination also has been associated with increased risk for all-cause mortality.

Our cultural fascination with physical beauty, as perpetually reinforced by the mass media and entertainment industries, contributes to the structural stigmatization of those with visible disfigurement (Griffiths and Mullock, 2018; Sarwer and Spitzer, 2021). Physically beautiful faces and bodies dominate the screens of movie theaters, televisions, and hand held devices. Most individuals are susceptible to engaging in social comparison to these images resulting in increased body image dissatisfaction for the viewer (Sperry et al., 2009). When those with visible disfigurement are represented in the media, they are more often or not in the role of the evil enemy—from Disney films to the James Bond movie franchise (Sarwer, 2021). These depictions reinforce the “what is anomalous is bad” stereotype.

Stigma and discrimination against those with visible disfigurement is also common (Swift and Bogart, 2021). Adults with facial or body disfigurement have been found to suffer from stigmatization in social situations, including friendships and romantic relationships, as well as overt discrimination in employment (Porter et al., 1986; Koster and Bergsma, 1990; Stone et al., 1992; Ginsburg and Link, 1993; Sarwer et al., 1998; Thompson and Kent, 2001; Horgan and MacLachlan, 2004; Rumsey and Harcourt, 2004; Tartaglia et al., 2005; Saradjian et al., 2008; Wisely and Gaskell, 2012; Mathias and Harcourt, 2014; Sharratt et al., 2018). Almost two-thirds of adults with a facial disfigurement reported avoidance of social situations and 71% of individuals reported that others had not wanted to become romantically involved with them because of their appearance (Broder et al., 2012). Almost 50% of these adults reported that their appearance had affected whether they had been hired for a job (Wisely and Gaskell, 2012).

## Application to the evaluation of candidates for vascularized composite allotransplantation

Vascularized composite allotransplantation (VCA) holds great promise for individuals with profound disfigurement and who have not had an acceptable response to the current generation of reconstructive procedures or prosthetics (Siemionow et al., 2009; Hautz et al., 2011; Pomahac et al., 2011; Khalifian et al., 2014; Dean and Talbot, 2017; Siemionow, 2017). To date, over 100 of these procedures have been performed worldwide. Many results have been quite impressive. Presently, active programs of research are investigating advances in surgical treatment and immunosuppression for persons who undergo these procedures (Siemionow et al., 2009; Pomahac et al., 2011; Siemionow, 2017). Similarly, the Chauvet Group, an international group of mental health and medical professionals, is considering the mental health issues in persons who undergo VCA (Kumnig et al., 2014; Jowsey-Gregoire et al., 2016; Kumnig and Jowsey-Gregoire, 2016).

While there is enthusiasm that VCA holds great potential for individuals who experience severe traumatic injuries, as well as those who have lost limbs secondary to systemic illness such as sepsis or cancer, little is known about the psychosocial issues of individuals who may be potential candidates. Many suffer with PTSD, mood, and anxiety disorders; others may overuse substances to address issues of physical or psychological pain. Issues with self-esteem, quality of life, and body image dissatisfaction are common. The psychosocial burden may be so severe that it contraindicates a VCA procedure. Anecdotal reports suggest that approximately one-third of patients who have approached established VCA programs around the United States have not undergone a procedure because of concerns about their psychiatric status. Other reports suggest that a subset of patients who have undergone VCA have experienced a failure of the procedure secondary to behavioral non-compliance (typically with immunosuppression medications) or have requested amputation of their hands.

Most VCA programs require patients to undergo a mental health evaluation as part of the preoperative evaluation process. While the Chauvet group and others have offered some guidance on the nature and structure of these evaluations, an established standard of care has yet to be established. The mental health professional, as well as all members of the VCA team, must consider not only issues of physical functioning, but the degree of psychosocial stress and likely benefit of a successful procedure (Caplan et al., 2019). Compliance with the postoperative immunosuppression treatments and other elements of care are likely psychologically challenging as well. Psychosocial status and functioning may leave one patient better suited for the postoperative challenges and threats to long-term success of the procedure than another. Resiliency is a likely pre-requisite, but is a notoriously difficulty psychological construct to predict and assess. As written by Butler and colleagues before the first VCA procedure was ever performed:

…it may be that people who have well-developed coping strategies and good social skills cope well with disfigurement, while those who find life generally more challenging, also cope poorly with disfigurement. The concern for us as clinicians…is that this group may also cope poorly with face transplantation; thus, the very group who might benefit most are those who are least likely to cope…., particularly if the results fall short of their expectations (Butler et al., 2004, p. 17).

Candidates and family members may believe that a successfully performed VCA procedure is critical to a new, post-injury life. Yet, the full extent of interest in and attitudes toward VCA among veterans and active military men and women is still largely unknown. The reality is that many individuals with profound disfigurements lead meaningful lives; some dedicate themselves to improving the treatment of others with these types of injuries.

As is the case for other forms of transplantation, support from family members, health care providers, and close friends is key to successful post-VCA adaptation. Patients’ social and health networks can also support and weigh in on the decision to pursue VCA as a treatment option. Additional research is needed to understand the nuances of patient, provider, and caregiver attitudes and perspectives on the VCA decision. Caplan et al. (2019) have suggested that patient advocates can provide an additional layer of protection for VCA candidates struggling with mental health issues and considering the risks and benefits of the procedure. Such advocates also can help VCA recipients challenged by compliance with the intense requirements of postoperative medical care.

## Conclusion and future directions

Traumatic injuries leave hundreds of thousands of individuals with permanent changes in physical functioning and appearance annually. Multidisciplinary care and treatment can help improve physical functioning, but many individuals experience permanent changes in physical appearance. Some experience untoward reactions to the traumatic event; others struggle with mood and anxiety. Self-esteem, body image, and quality of life is negatively impacted for many. Those with visible disfigurement are routinely stigmatized in a range of interpersonal situations and some experience outright discrimination from others. The psychosocial burden of living with disfigurement in a society where physical beauty is so idealized simply cannot be overstated.

For some individuals with profound disability and disfigurement, VCA offers a new treatment option. Literature on the surgical treatment and medical management of patients who undergo VCA procedures is growing. Understanding of the psychosocial issues of persons who are potential candidates for VCA is still in its early stages, with a limited number of case reports and expert opinion articles in the literature. The relationship between impairments in physical functioning, the psychosocial status and functioning of potential candidates, and their appropriateness for a VCA procedure remains poorly understood.

Robust, hypothesis-driven research on the psychological comorbidities of individuals who have suffered injuries that would potentially make them appropriate for VCA procedures would provide critically important information that could inform who might best benefit from VCA. This work would allow thought leaders to provide more specific information on the psychosocial factors which affect candidacy for VCA. Such information can be used to refine the psychosocial patient selection criteria and its role in optimizing postoperative outcomes. Subsequent standards of care can then be articulated and ensure that candidates for VCA procedures at programs around the world are being appropriately evaluated and treated as they undergo these profoundly life enhancing procedure, and, ideally, lessen the psychosocial burden of living with a visible disfigurement.

## Author contributions

DS wrote the initial draft of the manuscript. LS, HG, and JS wrote and revised sections of the manuscript. All authors subsequently read, revised and approved the submitted version.
